# UBASH3B Is a Novel Prognostic Biomarker and Correlated With Immune Infiltrates in Prostate Cancer

**DOI:** 10.3389/fonc.2019.01517

**Published:** 2020-01-15

**Authors:** Zijun Wang, Yinhuai Wang, Mou Peng, Lu Yi

**Affiliations:** ^1^Department of Dermatology, The Second Xiangya Hospital, Central South University, Changsha, China; ^2^Department of Urology, The Second Xiangya Hospital, Central South University, Changsha, China

**Keywords:** UBASH3B, prostate cancer, prognosis, tumor-infiltrating immune cells, ceRNA network

## Abstract

**Background:** UBASH3B (STS1) is an important gene that negatively regulates T-cell receptor signaling in activated T-lymphocytes that involved in cancers. However, the function of UBASH3B in prostate cancer (PCa) and the correlation between UBASH3B and tumor-infiltrating immune cells still remain unclear.

**Methods:** Real-time PCR and immunohistochemistry were applied to detect mRNA and protein expression of UBASH3B in PCa patients and benign prostate hyperplasia patients (BPH). Clinical features of patients with PCa were recorded and Kaplan Meier curve was subsequently plotted. Based on mRNA expression of UBASH3B, patients with PCa from TCGA database were divided into low-UBASH3B-expression group and high-UBASH3B-expression group for construct lncRNA-miRNA-mRNA network and analyzing GO and KEGG pathways. Single gene analysis method was performed by using GSEA to interpret gene expression data in PCa. The PPI network was constructed using STRING and the correlation between UBASH3B and tumor-infiltrating immune cells was analyzed by TIMER and CIBERSORT.

**Results:** The mRNA and protein expression of UBASH3B were upregulated in PCa. The abundant expression of UBASH3B is associated with poor prognosis in PCa. The subnetwork of UBASH3B contains three lncRNAs (MIAT, LINC01297, MYLK-AS1) and four miRNAs (hsa-miR-200a-3p, hsa-miR-455-5p, hsa-miR-192-5p, hsamiR- 215-5P). The mRNA expression of UBASH3B was involved in 28 KEGG pathways. GSEA analysis showed that 18 hallmark gene sets were significantly enriched in high-UBASH3B-expression, whereas 1 gene set was enriched in low-UBASH3B-expression. PPI network revealed a tightly interaction between UBASH3B and LCP2 (an immune related gene). TIMER and CIBERSORT database indicated that UBASH3B was correlated with 11 types of tumor-infiltrating immune cells (naïve B cell, memory B cells, resting CD4^+^ memory T cell, activated CD4^+^ memory T cell, regulatory T cell, activated NK cell, M2 macrophages, resting dendritic cells, activated dendritic cells, resting mast cells, neutrophils).

**Conclusions:** In conclusion, UBASH3B may be a novel potential prognostic biomarker and is associated with tumor-infiltrating immune cells in tumor microenvironment, suggesting UBASH3B as a potential target for future treatment of PCa.

## Introduction

Prostate cancer (PCa) is the most common male malignant diseases in Western industrialized countries. In 2018, 164,690 cases of prostate cancer were newly diagnosed, and 29,430 cases of death were recorded in the United States (1). Despite the increasing numbers of PCa patients identified after prostate-specific antigen (PSA) screening, many cases have been initially diagnosed as metastatic cancer. Unfortunately, there is no opportunity for surgical treatment for these patients. Even if patient underwent surgical treatment, patients who did not find a micrometastatic mass before surgery would relapse. It seems that surgical treatment is difficult to achieve the effect of radical resection, thus patients have been suffering economic losses and pain all the time. It is important to identify potential micrometastases prior to surgery in order to be able to avoid surgery in PCa patients. Currently, the mechanisms of tumorigenesis and metastasis are still unclear, and biomarkers for the diagnosis of prostate cancer are urgently needed.

Previous studies have reported several diagnostic and therapeutic biomarkers in prostate cancer, such as PCA3 (2) and TRPM8 as well as its isoform (3). Ubiquitin-associated and SH3 domain-containing B (UBASH3B) is involved in cancers pathogenesis and plays an important role in oncological biology by interacting with several molecules. UBASH3B negatively regulates protein kinase activity and peptidyl-tyrosine dephosphorylation and promotes tamoxifen resistance by ESR1 negative regulation in breast cancer (4). Aberrant expression of UBASH3B in triangle-negative breast cancer boosts tumor growth, invasion, and metastasis through EGFR regulation (5). In acute myeloid leukemia (AML), UBASH3B is upregulated by AML1-ETO fusion protein to promote the growth of AML cells by functionally regulating CBL (6). In addition to the above downstream pathways, several upstream pathways have been reported. miR-200a targets UBASH3B against invasion and is downregulated in triple-negative breast cancer (5). miR-148a-3p binds to UBASH3B and negatively regulates FcγRIIA mediated platelet activation (7). The mechanism of invasion and metastasis of UBASH3B in triple-negative breast is dependent on its tyrosine phosphatase activity (5). UBASH3B not only plays a critical role in the invasion and metastasis of cancer, but also affects the immune responses in cancer. UBASH3B also named Sts1 (an inhibitor of T cell receptor signaling-1), and negatively regulates T-cell receptor signaling in activated T lymphocytes via dephosphorylation of ZAP70, and regulates IFN-α-induced autophagy in B cells (8). This suggests that UBASH3B may be a potential biomarker for prostate cancer and is involved in the immune response in cancer. However, the function of UBASH3B and the correlation of UBASH3B and tumor-infiltrating immune cells remain unclear.

In this study, we investigated mRNA and protein expression of UBASH3B in prostate cancer. All patients were followed up to generate survival curves to assess the relationship between prognosis and UBASH3B expression levels. By constructing lncRNA-miRNA-mRNA and PPI networks, it was suggested that UBASH3B may interact with LCP2 and together participate in the immune response of tumor microenvironment. Our results suggested that UBASH3B may be a novel prognostic marker and was associated with tumor-infiltrating immune cells in prostate cancer.

## Materials and Methods

### Specimen Preparation

Thirty two samples from prostate cancer patients and 9 samples from benign prostatic hyperplasia (BPH) patients were surgically harvested from the Department of Urology, Second Xiangya Hospital of Central South University with the permission of the patients. All patients signed an informed consent form, which was approved by the Clinical Research Ethics Committee of the Second Xiangya Hospital of Central South University.

### Immunohistochemistry (IHC)

The patients' sample were fixed in 10% neutral buffered formalin for 24 h and then embedded in paraffin. Tissue slides were prepared and deparaffinized by baking in oven 60°C for 1 h. Then antigen unmasking was done in boiling container with sodium citrate buffer for 20 min. Goat serum was used for blocking. Slides were then stained with UBASH3B antibody (1:500, Sigma, HPA038605) overnight at 4°C and secondary antibodies were incubated for 1 h at room temperature. After adding substrate and hematoxylin staining, slides were covered and observed by microscope. The expression density of UBASH3B in IHC tissue was evaluated and divided into two group: low expression group, including negative (–) and weak (+) staining, high expression group, including moderate (++) and strong (+++) staining (9).

### Quantitative Real-Time PCR

Fresh frozen samples were used for real-time PCR. Total RNA was extracted with Trizol reagent according to the manufacturer's protocol (Thermo Fisher Scientific). Reverse transcription reaction was performed using random hexamers and SuperScript-III (Thermo Fisher Scientific). qPCR was cycled with the ABI 7900 real-time PCR system using SYBR Green PCRMix. The amplification efficiency was evaluated by the standard curve. Primers for UBASH3B are followed from 5′ to 3′: ttatgtgcgaggacagcaag(F), ctctgcagcaaagtcagcag(R). Primers of β-actin are followed from 5′ to 3′: cattaaggagaagctgtgct(F), gttgaaggtagtttcgtgga(R). This experiment was repeated three times.

### Follow-Up

Patients with prostate cancer receive a follow-up every 3 months for the first 2 years, and follow-up every 6 months for the next 2 years, followed by annual follow-up. Death events and death times were recorded. Overall survival was determined based on the time from surgical operation to death or the last follow up. For patients who were absent at the last follow-up, we conducted a telephone follow-up.

### lncRNA-miRNA-mRNA Network Construction

Prostate cancer datasets including RNA-Seq and miRNA from the cancer genome atlas (TCGA-PRAD) were downloaded. The data from TCGA-PRAD was constructed into a matrix. Ensemble names of genes were transited into symbol names using Microsoft of R version 3.5.1. Patients were divided into two groups: low UBASH3B expression and high UBASH3B expression. Differentially expressed genes were identified by Package GDCRNATools and differentially expressed lncRNAs were selected by “edgeR” method (fold change more than 1.8 and *p* < 0.01). The miRcode and mirTarBase databases were utilized to pair lncRNA-miRNA and mRNA-miRNA, respectively. Nodes and edges files were exported and visualization was performed by Cytoscape 3.6.1. Volcano map and heatmap of both mRNAs and lncRNAs were plotted and Gene Ontology (GO) and KEGG pathway were investigated.

### Gene Set Enrichment Analysis (GSEA)

In the TCGA-PARD database, 499 prostate cancer cases were divided into two expression level groups according to the median expression value of UBASH3B. GSEA was then performed to detect the gene sets that were enriched in the gene rank in the two groups for identifying potential hallmark of prostate cancer. The annotated gene sets of h.all.v6.2.symbols.gmt in the Molecular Signatures Database (MSigDB) was selected in GSEA version 3.0. We performed 1,000 times of permutations. Collapse dataset to gene symbols was “False.” The permutation type was “phenotype.” GSEA were run and the cut-off criteria were as follows: normalized enrichment scores (NES) > 1.0, false discovery rate (FDR) *q* > 0.25 and nominal *p* < 0.05. We chose all hallmark gene sets with significant enrichment and displayed gene sets enrichment plots. Then 100 most significant correlation hub genes in these gene sets was imported to construct PPI network.

### Protein-Protein Interaction Network (PPI)

The online tool of searching tool for the retrieval of interacting genes (STRING, https://string-db.org) was used for PPI network construction and hub genes screening. Multiple protein model was selected and 100 most significant correlation hub genes were input to search. PPI network was constructed by setting medium confidence at 0.400. Then the irrelevant genes were excluded and 19 genes were used for constructing the PPI network, then heatmap of these 19 genes was plotted. The correlations between UBASH3B and LCP2, or PIK3CG, or BIRC3 were evaluated and displayed.

### Comprehensive Correlation Analysis in Tumor-Infiltrating Immune Cells

A website called tumor immune estimation resource [TIMER (10), https://cistrome.shinyapps.io/timer] served for analysis of tumor-infiltrating immune cells, and the correlations between the infiltrating level of different subsets of immune cells and UBASH3B or LCP2 or PIK3CG or BIRC3 were computably detected. The immune cells contained CD4^+^ T cells, CD8^+^ T cells, B cells, macrophages, neutrophils, and dendritic cells. For further investigation, TPM data of RNA-seq was converted from FPKM data and used for estimating the abundance of different immune cell types in tumor microenvironment by CIBERSORT (11) (https://cibersort.stanford.edu/). RNA-seq data of 499 prostate cancer samples were divided into two groups: Low UBASH3B expression and High UBASH3B expression, according to the expression level of UBASH3B. Data were imported into CIBERSORT and LM22, including 22 immune cell types, selected as signature gene file. The Mixture file was made with gene symbol and sequencing values. One thousand was set as permutation value for statistical analysis. Disable quantile normalization was selected. Relative fractions of each immune cell types in each sample were calculated and Graphpad Prism 7 was used for analysis and plotting.

### Statistical Analysis

The measurement data are displayed as the mean ± SD. Unpaired *t*-test was used for analyzing statistical assessments. The association between UBASH3B and clinical characteristic variables was analyzed using Pearson chi-squared test or Fisher's exact test. Graphpad prism 7 was utilized for Kaplan-Meier survival analyses using the log-rank test. The Cox proportional hazards regression model was used for univariate and multivariate analyses, and statistically significant difference was considered when *P* < 0.05.

## Results

### UBASH3B Is Upregulated in Prostate Cancer

**Figure 1:**
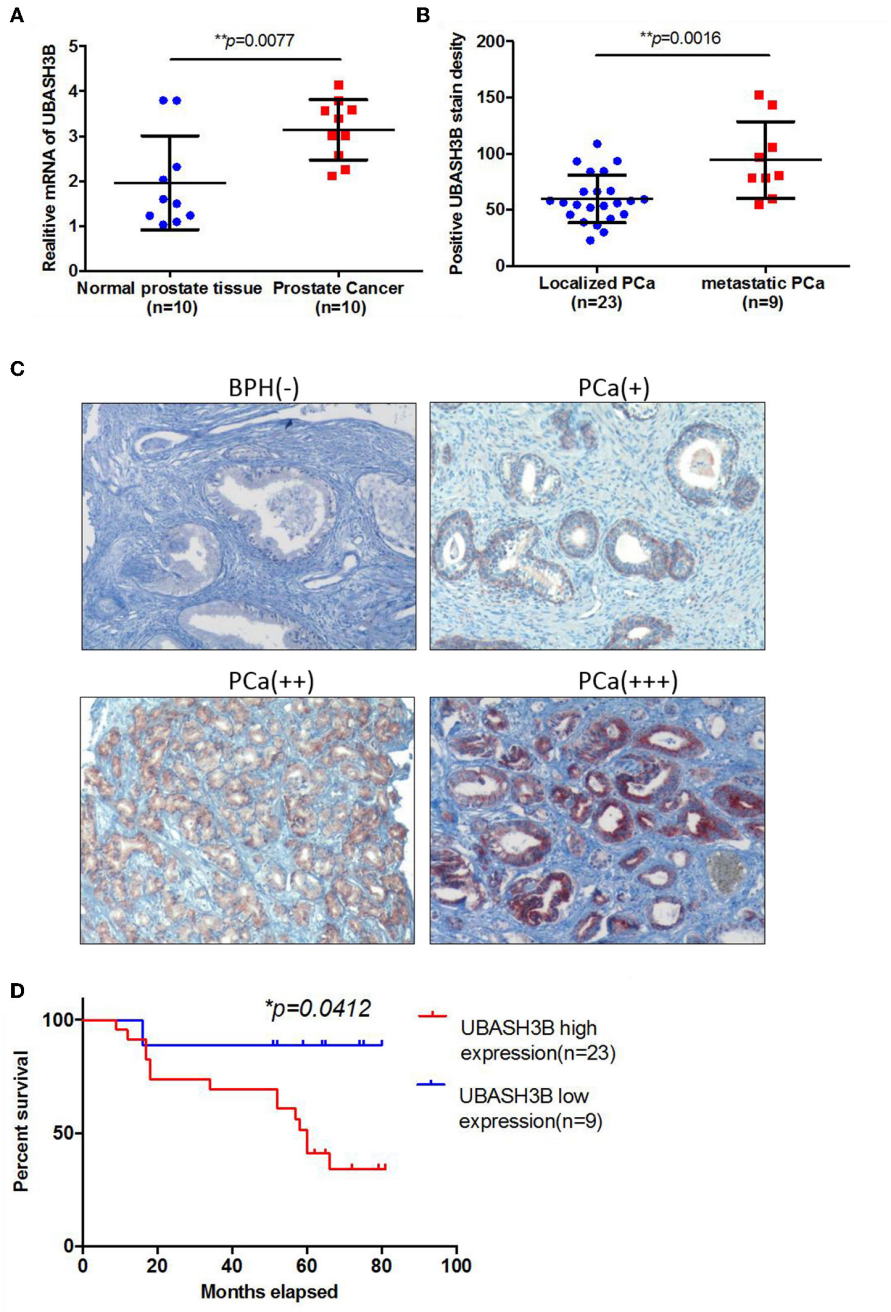
**(A)** The mRNA expression of UBASH3B is significantly upregulated in prostate cancer tissue as compared with normal prostate tissue (*p* = 0.0077). **(B)** The expression of UBASH3B is significantly associated with tumor metastatic status of prostate cancer (*p* = 0.0016). **(C)** Differential expression level of UBASH3B in benign prostate hyperplasia (BPH) tissue and prostate cancer (PCa) tissue. Weak expression (+); Moderate expression (++); Strong expression (+ + +). **(D)** Data indicate that the expression levels of UBASH3B are associated with poor prognostic survival in prostate cancer patients (*p* = 0.0412).

To investigate the mRNA expression level of UBASH3B in PCa and BPH tissues, real-time PCR was used and UBASH3B was found to be significantly upregulated in PCa tissues in comparison with BPH tissues (*P* = 0.0077) (Figure 1A). We performed immunohistochemistry staining for UBASH3B expression in prostate cancer and BPH tissues. Quantitative analysis of IHC found that the expression of UBASH3B was significantly upregulated in the prostate cancer tissues compared with BPH. UBASH3B expression was further found to be higher in PCa patients with metastasis than localized PCa patients (Figure 1B). Differential expression levels of UBASH3B in BPH and PCa tissue were displayed in (Figure 1C). To further investigate the correlation between UBASH3B expression level and disease progression, we explored the relationship between expression level of UBASH3B and clinical characteristics in 32 patients (Table 1). No correlation was revealed between UBASH3B expression level and age, TNM stage, metastasis, Gleason score, or total PSA. Kaplan-Meier analysis suggested that prostate cancer patients with high UBASH3B expression level experienced a significantly shorter overall survival of 5-year than those with low UBASH3B levels (*p* = 0.042) (Figure 1D). Univariate and multivariate COX regression analysis indicated UBASH3B was associated with poor survival but was not an independent prognostic factor (Table 2).

**Table 1:** Relationship between UBASH3B expression level and clinicopathological variables and in prostate cancer patients.

**Classification**	**Total**	**Low UBASH3B expression**	**High UBASH3B expression**	***p***
**AGE(YEAR)**				
< 60	4	1	3	1.000
≥ 60	28	8	20	
**TNM STAGE**				
T1–T2	20	6	14	1.000
T3–T4	12	3	9	
**METASTASIS**				
–	23	7	16	1.000
+	9	2	7	
**GLEASON SCORE**				
≤ 6	7	4	3	0.191
7	13	2	11	
≥ 8	12	3	9	
**TOTAL PSA**				
≤ 20 ng/ml	5	1	4	1.000
>20 ng/ml	27	8	19	

*Low expression including no (–) and weak (+) staining*.

*High expression including moderate (++) and strong (+++) staining*.

*Metastasis including lymph node and distant metastasis*.

**Table 2:** Univariate and multivariate regression analysis to determine the independent prognostic factor.

**Variables**	**Univariate**			**Multivariate**		
	**Coef**	**HR(95%CI)**	***P***	**Coef**	**HR(95%CI)**	***P***
Age	0.083	1.1 (1–1.2)	0.02	0.07	1.07 (0.99–1.17)	0.08
tPSA	−0.41	0.67 (0.18–2.4)	0.53	−0.55	0.58 (0.13–2.48)	0.46
TNM	0.57	1.8 (0.63–4.9)	0.28	0.58	1.78 (0.17–17.9)	0.62
UBASH3B	1.8	6.3 (0.83–48)	0.04	1.3	3.65 (0.44–30.7)	0.23
Gleason score	0.19	1.2 (0.62–2.4)	0.58	0.19	1.21 (0.58–2.52)	0.61
Metastasis status	0.72	2.1 (0.73–5.8)	0.17	0.79	0.79 (0.06–9.95)	0.86

### lncRNA-miRNA-mRNA Network Construction

**Figure 2:**
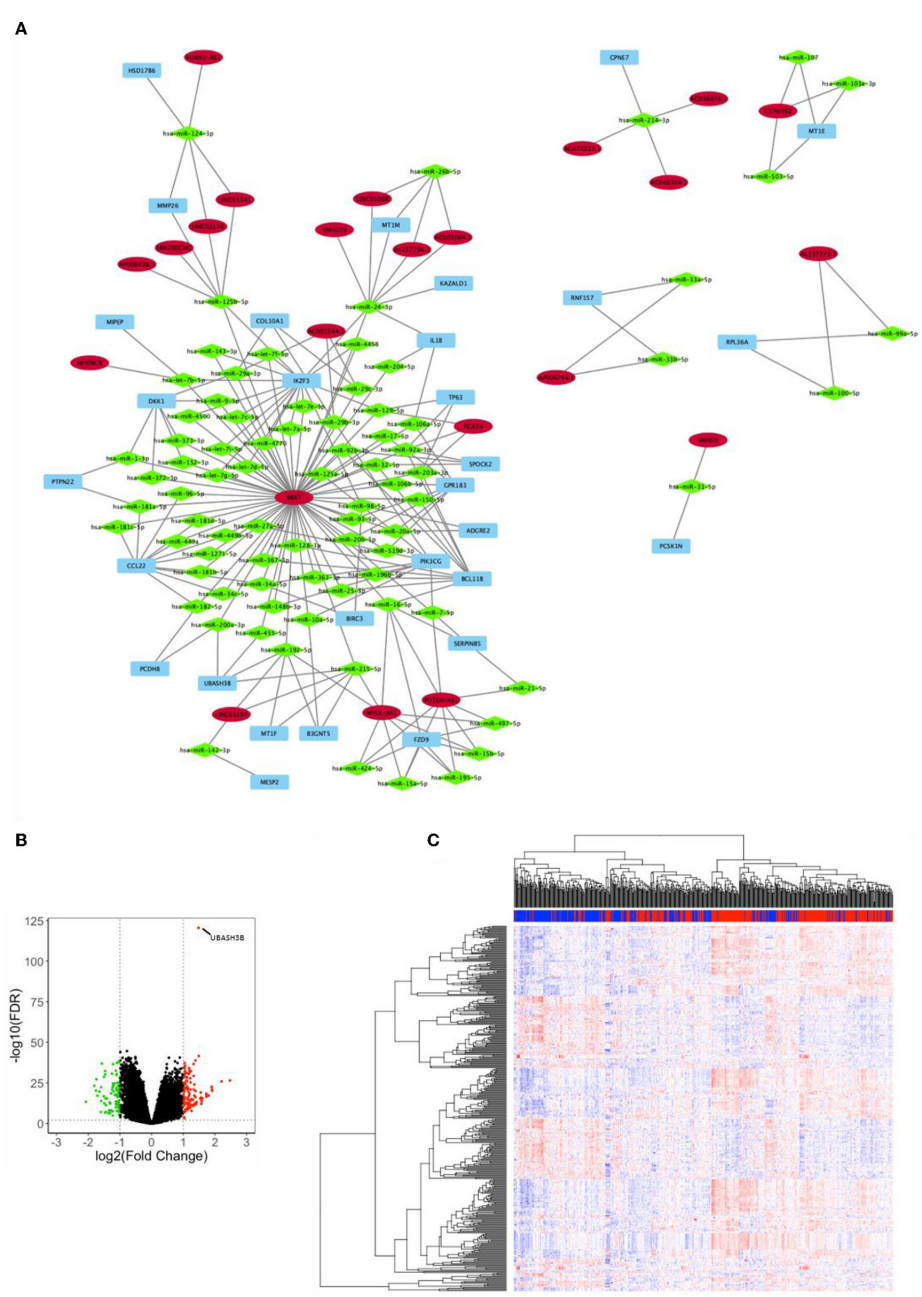
**(A)** lncRNA-miRNA-mRNA network was constructed from R package of GDCRNATools according to different UBASH3B expression levels and visualized by microsoft Cytoscape. Blue, mRNA; Green, miRNA; Red, lncRNA. **(B)** Volcano plot of differentially expressed mRNAs and lncRNAs is based on differences in expression levels of UBASH3B. **(C)** Heatmap of mRNAs and lncRNAs in TCGA-PRAD according to different UBASH3B expression level (Blue, samples with low UBASH3B expression; Red, samples with high UBASH3B expression).

Analysis for differentially expressed lnRNA and mRNA were performed using “edgeR.” Three hundred four differentially expressed mRNAs and 55 differentially expressed lncRNAs were calculated to find shared miRNAs for “nodes” and “edges” files. We obtained 134 nodes and 227 edges and utilized to construct lncRNA-miRNA-mRNA network (Figure 2A). The subnetwork of UBASH3B contained 3 lncRNAs (MIAT, LINC01297, MYLK-AS1) and 4 miRNAs (hsa-miR-200a-3p, hsa-miR-455-5p, hsa-miR-192-5p, hsa-miR-215-5p). Volcano map of both mRNA and lncRNA showed up-regulated and down-regulated genes (Figure 2B). Then heatmap was shown in Figure 2C.

### GO and KEGG Pathway

**Figure 3:**
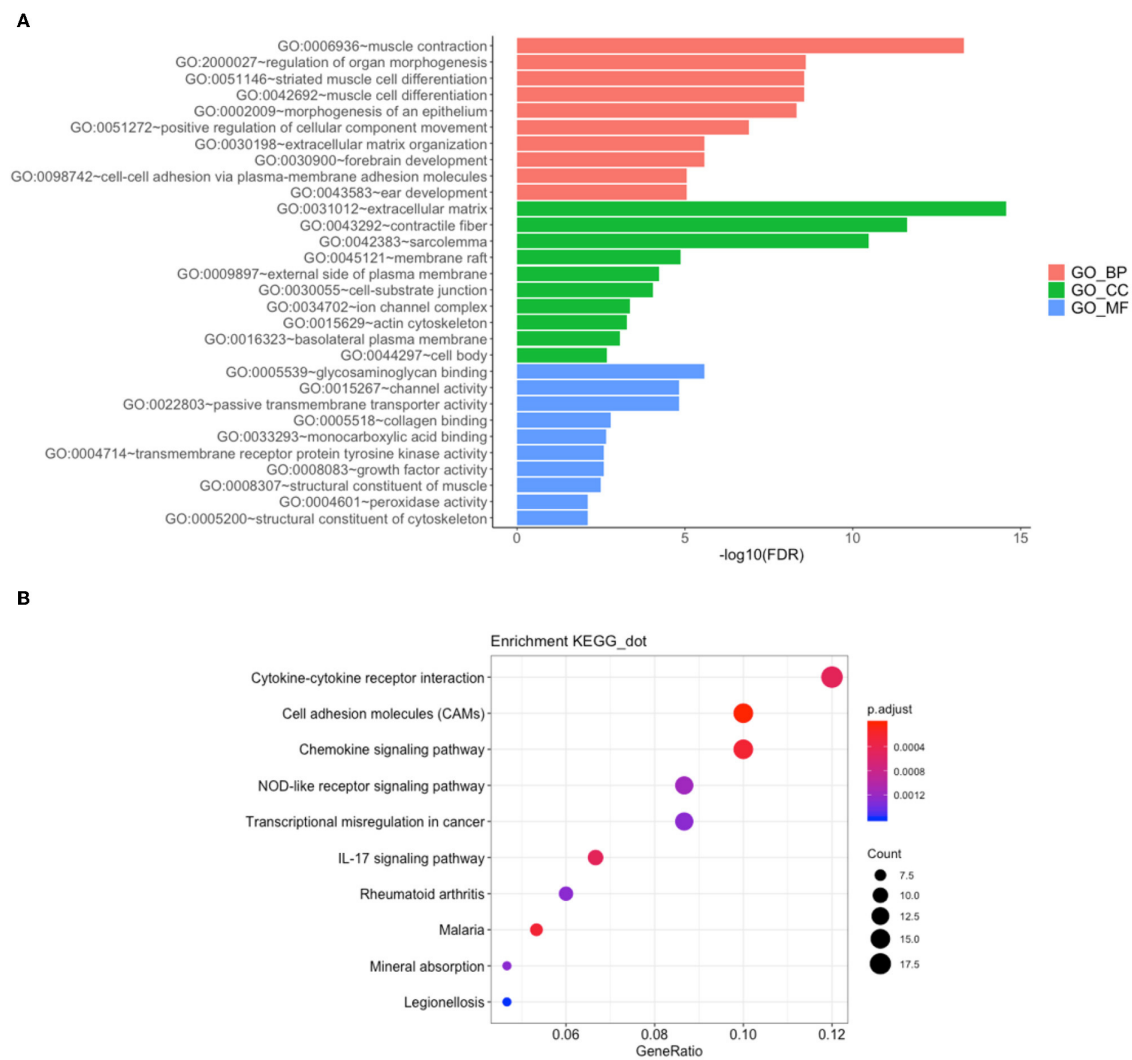
**(A)** Gene Ontology (GO) have been analyzed and shows the Top 10 of BP, CC, and MF(BP, Biological Process; CC, Cellular Component; MF, Molecular Function). **(B)** KEGG pathways is analyzed and the Top 10 pathway is mapped according to the differential expression level of UBASH3B.

Gene ontology contains biological process, cellular components, and molecular function. The top 10 of each aspect were shown in Figure 3A. The enriched biological process involved in GO: 0006936:muscle contraction, GO:2000027:regulation of organ morphogenesis, GO:0051146:striated muscle cell differentiation, GO: 0042692:muscle cell differentiation, GO: 0002009: morphogenesis of an epithelium, GO: 0051272: positive regulation of cellular component movement, GO: 0030198: extracellular matrix organization, GO: 0030900:forebrain development, GO: 0098742: cell-cell adhesion via plasma-membrane adhesion molecules, GO: 0043583: ear development. The enriched cellular components involved in GO: 0031012: extracellular matrix, GO: 0043292: contractile fiber, GO: 0042383: sarcolemma, GO: 0045121: membrane raft, GO: 0009897: 0030055: cell-substrate junction, GO: 0034702: ion channel complex, GO: 0015629: actin cytoskeleton, GO: 0016323: basolateral plasma membrane, GO: 0044297: cell body. The enriched molecular function involved in GO:0005539: glycosaminoglycan binding, GO: 0015267: channel activity, GO: 0022803: passive transmembrane transporter activity, GO: 0005518: collagen binding, GO: 0033293: monocarboxylic acid binding, GO: 0004714: transmembrane receptor protein tyrosine kinase activity, GO: 0008083: growth factor activity, GO: 0008307: structural constituent of muscle, GO: 0004601: peroxidase activity, GO: 0005200: structural constituent of cytoskeleton. There were 28 KEGG pathways involved in variations of UBASH3B expression (Table 3) and top 10 pathways were displayed in Figure 3B. The top 10 pathways are cytokine-cytokine receptor interaction, cell adhesion molecules (CAMs), chemokine signaling pathway, NOD-like receptor signaling pathway, transcriptional misregulation in cancer, IL-17 signaling pathway, Rheumatoid arthritis, Malaria, Mineral absorption, Legionellosis.

**Table 3:** KEGG pathway involved in PRAD according to UBASH3B expression level.

**ID**	**Description**	**GeneRatio**	**BgRatio**	**p.adjust**
hsa04514	Cell adhesion molecules (CAMs)	15/150	144/7840	1.77E-05
hsa04062	Chemokine signaling pathway	15/150	190/7840	0.000238547
hsa05144	Malaria	8/150	49/7840	0.000238547
hsa04657	IL-17 signaling pathway	10/150	93/7840	0.000477884
hsa04060	Cytokine-cytokine receptor interaction	18/150	294/7840	0.000477884
hsa04621	NOD-like receptor signaling pathway	13/150	178/7840	0.001155184
hsa04978	Mineral absorption	7/150	51/7840	0.001280394
hsa05202	Transcriptional misregulation in cancer	13/150	186/7840	0.001280394
hsa05323	Rheumatoid arthritis	9/150	91/7840	0.001280394
hsa05134	Legionellosis	7/150	55/7840	0.001587716
hsa04640	Hematopoietic cell lineage	9/150	97/7840	0.001732903
hsa04512	ECM-receptor interaction	8/150	82/7840	0.002766479
hsa05410	Hypertrophic cardiomyopathy (HCM)	8/150	85/7840	0.003285433
hsa04668	TNF signaling pathway	9/150	110/7840	0.003582737
hsa05414	Dilated cardiomyopathy (DCM)	8/150	91/7840	0.004565533
hsa05133	Pertussis	7/150	76/7840	0.007632455
hsa04933	AGE-RAGE signaling pathway in diabetic complications	8/150	100/7840	0.007632455
hsa05167	Kaposi sarcoma-associated herpesvirus infection	11/150	186/7840	0.009718886
hsa04151	PI3K-Akt signaling pathway	16/150	354/7840	0.0126229
hsa04510	Focal adhesion	11/150	199/7840	0.015177136
hsa05150	*Staphylococcus aureus* infection	6/150	68/7840	0.01803509
hsa04672	Intestinal immune network for IgA production	5/150	49/7840	0.021119439
hsa05146	Amoebiasis	7/150	96/7840	0.021119439
hsa05142	Chagas disease (American trypanosomiasis)	7/150	103/7840	0.03011686
hsa05143	African trypanosomiasis	4/150	37/7840	0.043099315
hsa04670	Leukocyte transendothelial migration	7/150	112/7840	0.044096453
hsa05145	Toxoplasmosis	7/150	113/7840	0.044559068

### Enriched Gene Sets in Low- and High- UBASH3B Expression Group

**Figure 4:**
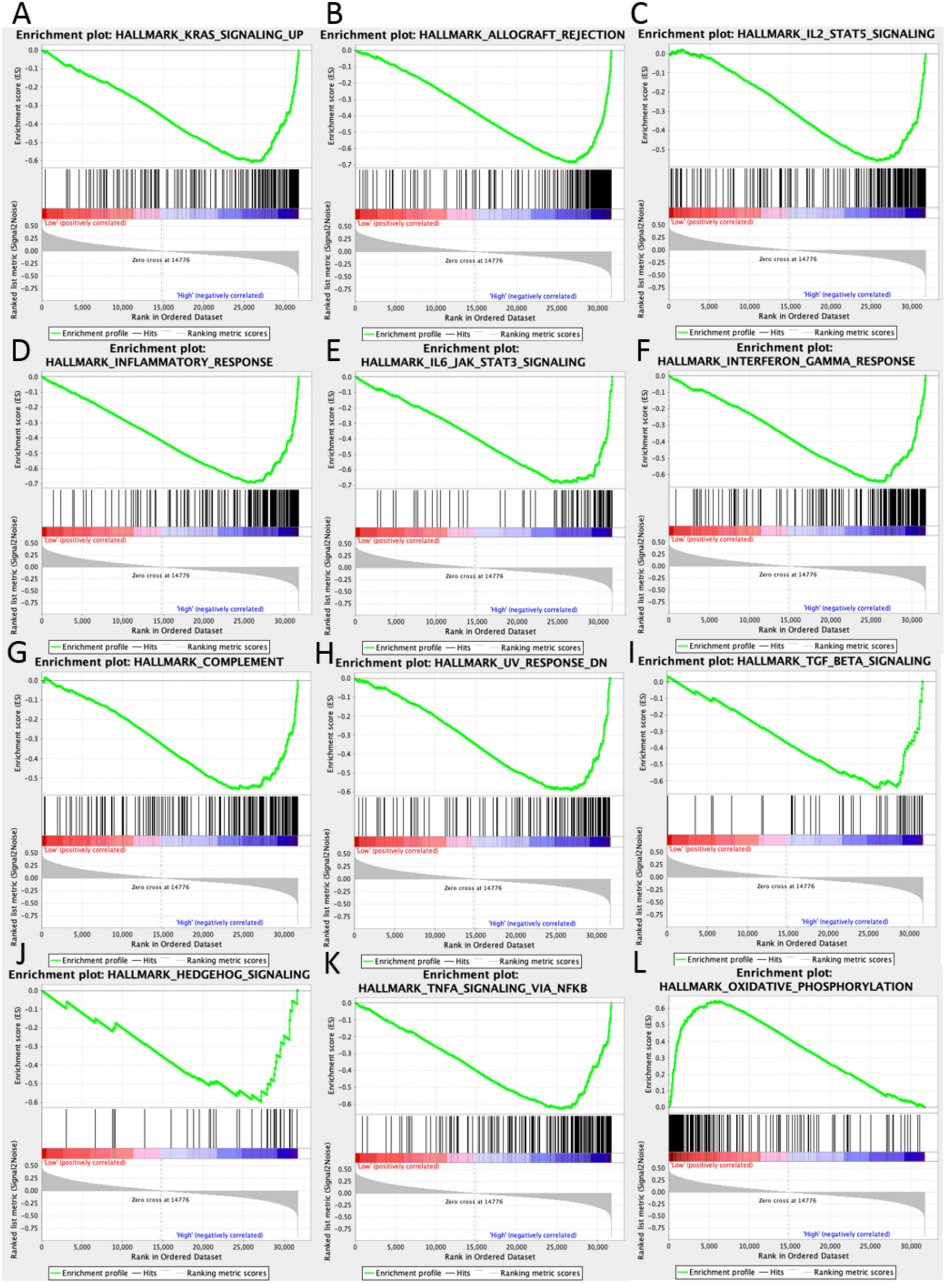
GSEA pathways using single-gene method of UBASH3B. **(A–K)** Up-regulated gene sets in the group of high UBASH3B expression. **(A)** Enrichment plot: HALLMARK_KRAS_SIGNALING_UP. **(B)** Enrichment plot: HALLMARK_ALLOGRAFT_REJECTION. **(C)** HALLMARK_IL2_STAT5_SIGNALING. **(D)** Enrichment plot: HALLMARK_INFLAMMATORY_RESPONSE. **(E)** Enrichment plot: HALLMARK_IL6_JAK_STAT3_SIGNALING. **(F)** Enrichment plot: HALLMARK_INTERFERON_GAMMA_RESPONSE. **(G)** Enrichment plot: HALLMARK_COMPLEMENT. **(H)** Enrichment plot: HALLMARK_UV_RESPONSE_DN. **(I)** Enrichment plot: HALLMARK_TGF_BETA_SIGNALING. **(J)** Enrichment plot: HALLMARK_HEDGEHOG_SIGNALING. **(K)** Enrichment plot: HALLMARK_TNFA_SIGNALING_VIA_NFKB. **(L)** Down-regulated gene sets in the group of high UBASH3B expression: Enrichment plot: HALLMARK_OXIDATIVE_PHOSPHORYLATION.

In high UBASH3B expression phenotype, 34 out of 50 gene sets were upregulated and 18 gene sets were significantly enriched at nominal *p* < 0.05, NES > 1.0 and FDR *q* > 0.25. The significantly upregulated hallmark gene sets involving in immune response were as follows: “KRAS SIGNALING UP,” “INFLAMMATORY RESPONSE,” “IL6 JAK STAT3 SIGNALING,” “IL2 STAT5 SIGNALING,” “COMPLEMENT,” “INTERFERON GAMMA RESPONSE,” “TGF BETA SIGNALING,” “TNFA SIGNALING VIA NFKB,” whereas gene sets involving in tumorigenesis and metastasis were “EPITHELIAL MESENCHYMAL TRANSITION,” “APOPTOSIS,” “MITOTIC SPINDLE,” “ANGIOGENESIS” in high UBASH3B expression group. All gene sets were shown in Table 4 and snapshot of enrichment results were shown in Figure 4.

**Table 4:** GSEA pathways up-regulated and down-regulated due to expression level of UBASH3B.

**GENE SETS**	**SIZE**	**NES**	**NOM p-val**	**FDR q-val**
**Upregulated gene sets in high UBASH3B expression group**				
HALLMARK_KRAS_SIGNALING_UP	193	−2.168	0.000	0.003
HALLMARK_INFLAMMATORY_RESPONSE	195	−2.147	0.002	0.002
HALLMARK_IL6_JAK_STAT3_SIGNALING	86	−2.070	0.002	0.004
HALLMARK_ALLOGRAFT_REJECTION	197	−2.027	0.004	0.006
HALLMARK_IL2_STAT5_SIGNALING	194	−2.019	0.002	0.005
HALLMARK_COMPLEMENT	194	−1.995	0.002	0.007
HALLMARK_TGF_BETA_SIGNALING	54	−1.967	0.004	0.010
HALLMARK_UV_RESPONSE_DN	140	−1.926	0.002	0.013
HALLMARK_INTERFERON_GAMMA_RESPONSE	197	−1.894	0.018	0.016
HALLMARK_HEDGEHOG_SIGNALING	35	−1.858	0.002	0.020
HALLMARK_APICAL_JUNCTION	191	−1.828	0.013	0.025
HALLMARK_TNFA_SIGNALING_VIA_NFKB	198	−1.818	0.025	0.026
HALLMARK_EPITHELIAL_MESENCHYMAL_TRANSITION	197	−1.787	0.032	0.030
HALLMARK_APOPTOSIS	158	−1.749	0.019	0.036
HALLMARK_MITOTIC_SPINDLE	197	−1.735	0.022	0.038
HALLMARK_ANGIOGENESIS	36	−1.659	0.031	0.062
HALLMARK_COAGULATION	132	−1.608	0.051	0.079
HALLMARK_APICAL_SURFACE	42	−1.603	0.031	0.077
**Downregulated gene sets in high UBASH3B expression group**				
HALLMARK_OXIDATIVE_PHOSPHORYLATION	183	1.803	0.032	0.180

### UBASH3B Is Correlated With LCP2, PIK3CG, and BIRC3 in Prostate Cancer

**Figure 5:**
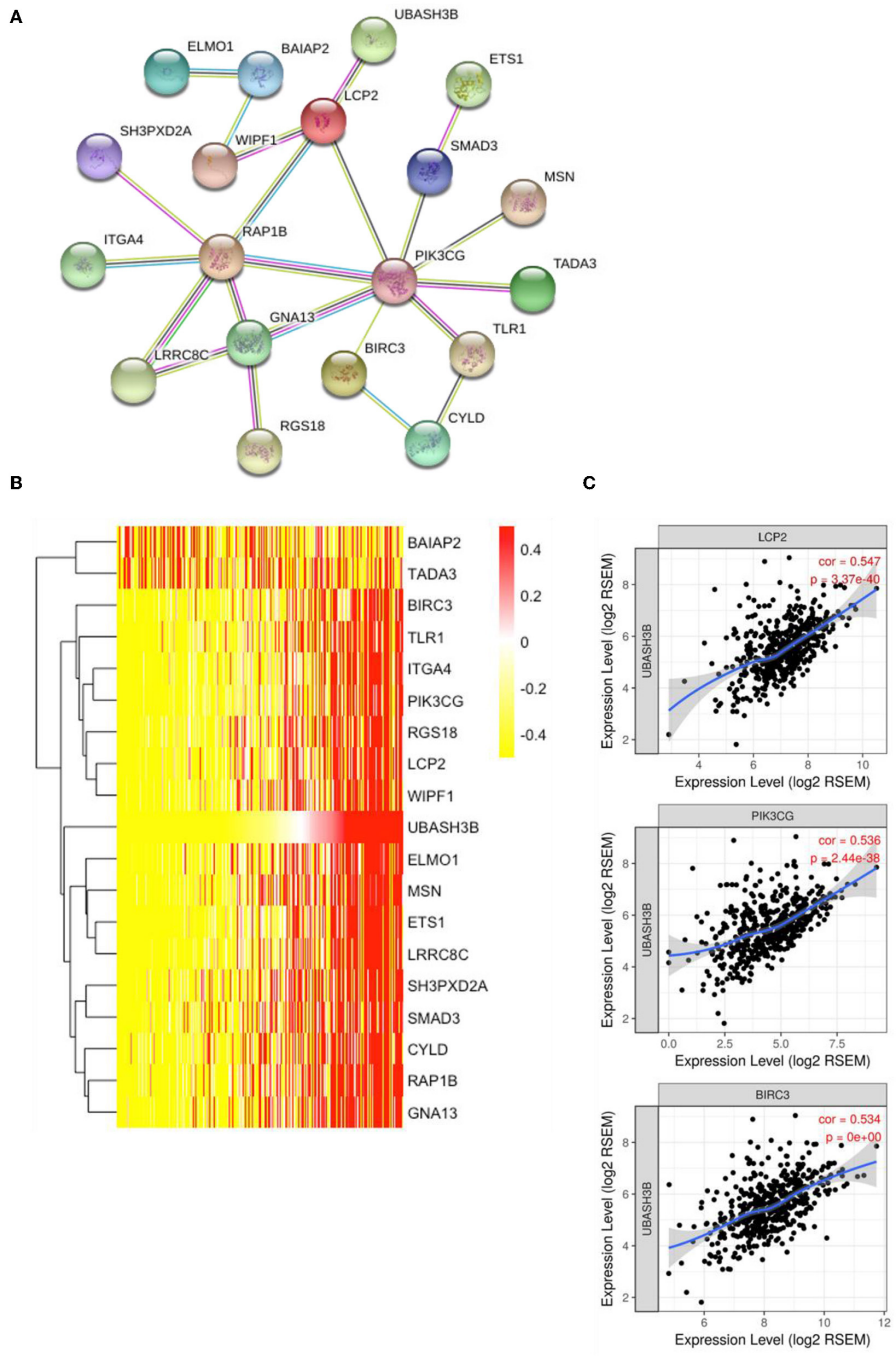
**(A)** The top 50 genes of each phenotype in the GSEA single-gene method was used to construct the PPI network by STRING (https://string-db.org/) and 19 genes was mapped (medium confidence equals 0.400). **(B)** Heatmap of 19 genes is involved in PPI network. **(C)** UBASH3B is significantly correlated with LCP2 (*p* = 3.37e-40, cor = 0.547), PIK3CG (*p* = 2.44e-38, cor = 0.536), and BIRC3 (*p* = 0, cor = 0.534) in prostate adenocarcinoma.

To construct PPI network, 100 UBASH3B correlated genes were analyzed and 19 genes participated in the network after elimination of disconnected node. It is suggested that LCP2 was an interactive protein of UBASH3B (Figure 5A). Heatmap of these 19 genes was displayed in Figure 5B. Furthermore, both PIK3CG and BIRC3 have been found in lncRNA-miRNA-mRNA network and PPI network. TIMER was used for correlation analysis and it indicated that UBASH3B was significantly correlated with LCP2 (*p* = 3.37e-40, cor = 0.547), PIK3CG (*p* = 2.44e-38, cor = 0.536), and BIRC3 (*p* = 0, cor = 0.534) in prostate cancer (Figure 5C).

### A Significant Correlation Between UBASH3B and Tumor-Infiltrating Immune Cells in Tumor Microenvironment

**Figure 6:**
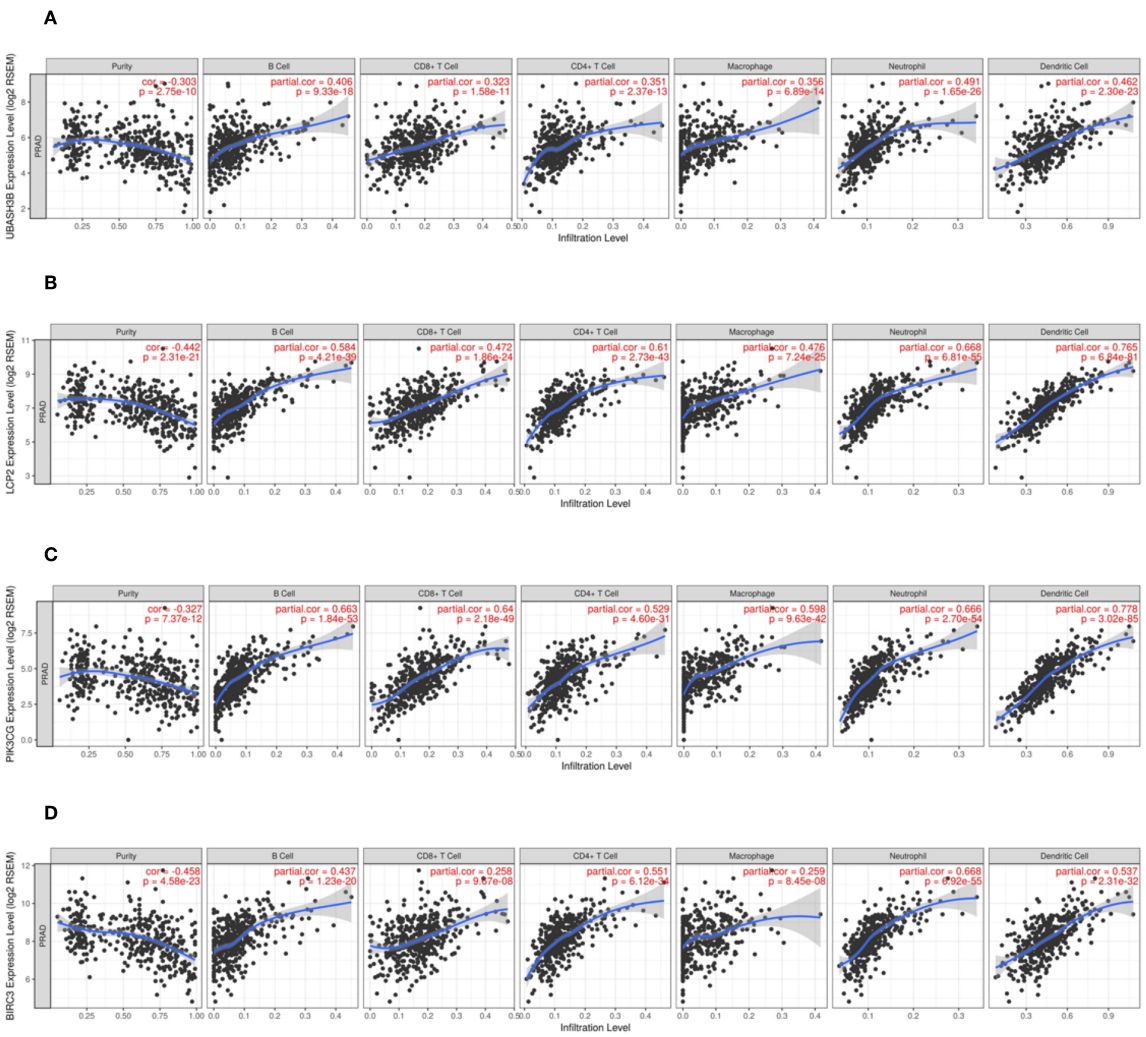
**(A)** There is a significant correlation between UBASH3B expression and immune cells, including CD4^+^ T cells, CD8^+^ T cells, B cells, macrophages, Neutrophils, and dendritic cells. **(B)** There is a significant correlation between LCP2 expression and immune cells, including CD4^+^ T cells, CD8^+^ T cells, B cells, macrophages, Neutrophils, and dendritic cells. **(C)** There is a significant correlation between PIK3CG expression and immune cells, including CD4^+^ T cells, CD8^+^ T cells, B cells, macrophages, Neutrophils, and dendritic cells. **(D)** There is a significant correlation between BIRC3 expression and immune cells, including CD4^+^ T cells, CD8^+^ T cells, B cells, macrophages, Neutrophils, and dendritic cells.

In order to know the relationship between UBASH3B expression and tumor-infiltrated immune cells, the tool of TIMER was used to analysis. There was a significant correlation between UBASH3B expression and different type of immune cells, including CD4^+^ T cells (*p* = 2.37e-13, cor = 0.351), CD8^+^ T cells (*p* = 1.58e-11, cor = 0.323), B cells (*p* = 9.33e-18, cor = 0.406), macrophages (*p* = 6.89e-14, cor = 0.356), Neutrophils (*p* = 1.65e-26, cor = 0.491), and dendritic cells (*p* = 2.30e-23, cor = 0.462) (Figure 6A). We also found that the UBASH3B-related genes, LCP2, PIK3CG and BIRC3 were correlated with a group of immune cells. For LCP2, CD4^+^ T cells (*p* = 2.73e-43, cor = 0.61), CD8^+^ T cells (*p* = 1.86e-24, cor = 0.472), B cells (*p* = 4.21e-39, cor = 0.584), macrophages (*p* = 7.24e-25, cor = 0.476), Neutrophils (*p* = 6.81e-55, cor = 0.668), and dendritic cells (*p* = 6.84e-81, cor = 0.765) were identified (Figure 6B). Further, the relationship between expression of PIK3CG and immune cells was described, including CD4^+^ T cells (*p* = 4.60e-31, cor = 0.351), CD8^+^ T cells (*p* = 2.18e-49, cor = 0.64), B cells (*p* = 1.84e-53, cor = 0.663), macrophages (*p* = 9.63e-42, cor = 0.598), Neutrophils (*p* = 2.70e-54, cor = 0.666) and dendritic cells (*p* = 3.02e-85, cor = 0.778) (Figure 6C). There was a significant correlation between BIRC3 expression and immune cells, including CD4^+^ T cells (*p* = 6.12e-34, cor = 0.551), CD8^+^ T cells (*p* = 9.67e-08, cor = 0.258), B cells (*p* = 1.23e-20, cor = 0.437), macrophages (*p* = 8.45e-08, cor = 0.259), Neutrophils (*p* = 6.92e-55, cor = 0.668), and dendritic cells (*p* = 2.31e-32, cor = 0.537) (Figure 6D). Altogether, it was suggested that UBASH3B may participate in immune response in tumor microenvironment through affecting immune cells.

**Figure 7:**
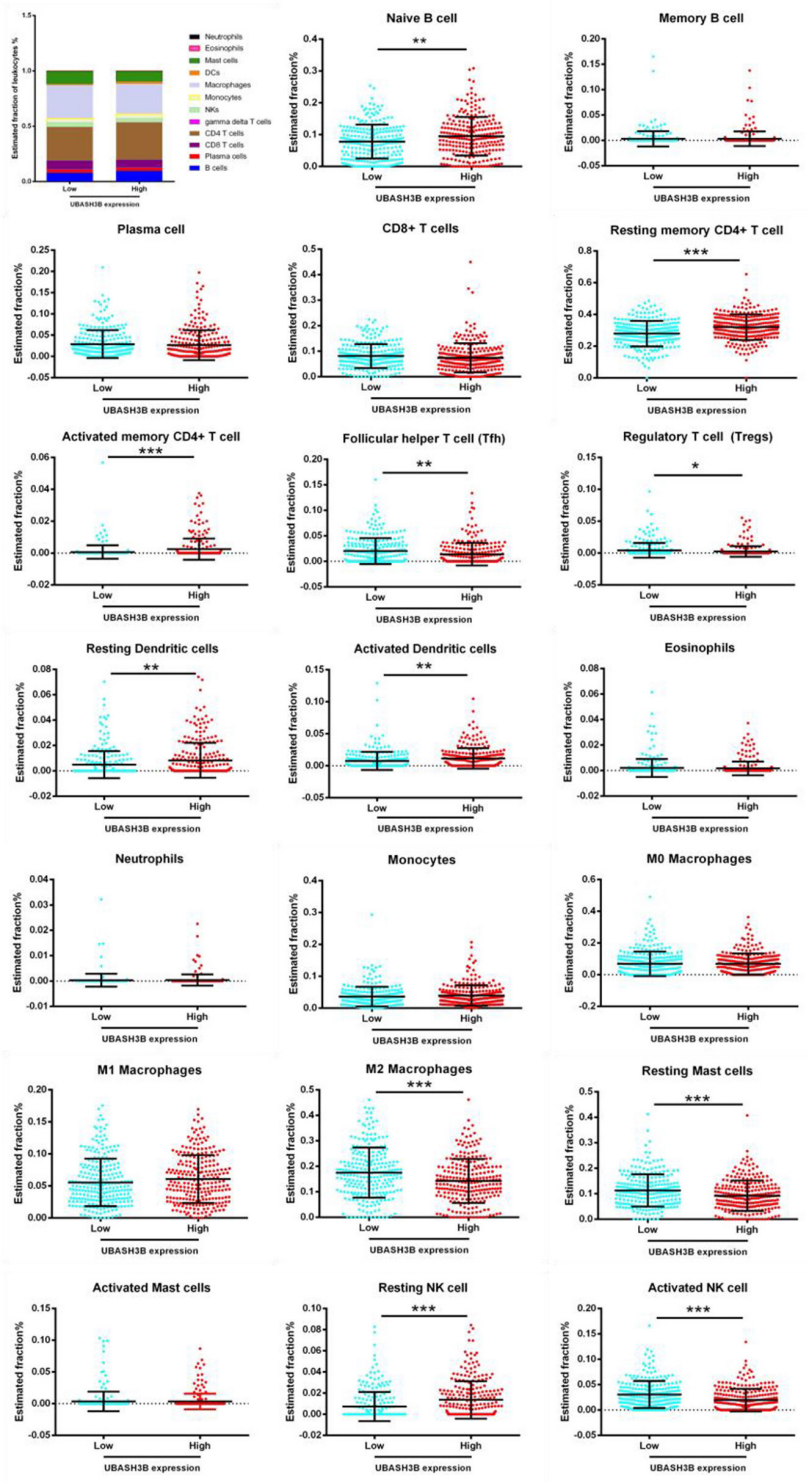
Based on TCGA-PRAD database, LM22 signature matrix is used for analysis using CIBERSORT online. Twenty-two kinds of tumor infiltrating immune cells are plotted according to UBASH3B expression level. There are significant differences in naïve B cell, memory B cells, resting CD4^+^ memory T cell, activated CD4^+^ memory T cell, regulatory T cell, activated NK cell, M2 macrophages, resting dendritic cells, activated dendritic cells, resting mast cells, neutrophils (***<0.001< **<0.01< *<0.05).

For further investigation, CIBERSORT analysis indicated that the UBASH3B expression was correlated with tumor-filtrating immune cells, including naïve B cell (*p* < 0.0001), memory B cells (*p* = 0.0424), resting CD4^+^ memory T cell (*p* = 0.0008), activated CD4^+^ memory T cell (*p* = 0.0274), regulatory T cell (*p* = 0.0368), activated NK cell (*p* = 0.0015), M2 macrophages (*p* = 0.0001), resting dendritic cells (*p* < 0.0001), activated dendritic cells (*p* = 0.0097), resting mast cells (*p* = 0.0009), neutrophils (*p* = 0.0499) (Figure 7). These altogether suggested that UBASH3B and its associated genes were important for immune cells infiltration in tumor pathology.

## Discussion

Prostate cancer is among the most common male genitourinary tumors worldwide. With the development of PSA screening and several treatment methods, patients with prostate cancer benefit from early diagnosis and intervention. However, in China, locally advanced prostate cancer accounts for only one-third of newly diagnosed prostate cancer patients, and the rest are diagnosed with invasive prostate cancer with advanced or metastatic symptoms and without surgical opportunities. Androgen deprivation therapy is the first-line treatment for hormone-sensitive prostate cancers with distal metastases. However, this carcinoma will eventually develop into castration-resistant prostate cancer (CRPC), and the therapeutic drugs will not obtain long-term remission, resulting in a poor prognosis. Therefore, it is necessary to further understand the drug-resistance related genes as a novel prognostic marker in prostate cancer. In this study, we identified UBASH3B as a novel potential prognostic biomarker for prostate cancer and investigated the correlation between UBASH3B and tumor-infiltrating immune cells in PCa.

UBASH3B, also known as STS1, TULA-2, or p70, is a member of the tyrosine phosphatase family of proteins that contain a ubiquitin-associated domain at the N-terminus, an SH3 domain, and a C-terminal domain with similarities to the catalytic motif of phosphoglycerate mutase. Functional studies linked UBASH3B expression to evaluate diseases states such as in thrombosis, leukemia, and triple-negative breast cancer. van der Meulen et al. have found that EGF receptor activation affects the interaction between UBASH3B and ShcA (12). UBASH3B plays a vital role through targeting Aurora B to microtubules in the timing and fidelity of chromosome segregation in human cells. UBASH3B also regulates myeloid proliferation through UBASH3B-CBL axis in human pre-leukemia (6). miR-148a-3p targets UBASH3B, which serves as a negative regulator of FcγRIIA-mediated platelet activation (7). UBASH3B is a functional target for drug-resistance and downregulated anti-invasive microRNA200a in triple-negative breast cancer (5). Importantly, the oncogenic potential of UBASH3B is dependent on its tyrosine phosphatase activity to promote invasion and metastasis. UBASH3B has been detected in prostate cancer cell lines and UBASH3B knockdown can decrease EGF-induced invasion and form tumor spheres in PC3 and DU145 cells (5). Real time PCR and western blot analysis results of our study showed mRNA and protein expression of UBASH3B were increased in prostate cancer samples as compared with that in benign prostatic hyperplasia samples. Kaplan-Meier survival analysis indicated that UBASH3B is a prognostic factor for 5-year survival in patients with prostate cancer. However, there was no significant relationship between UBASH3B expression and age/clinical stage/Gleason score/metastasis/total PSA. The high-risk status of the patients may affect the outcome of this study. The high-risk criteria of prostate cancer patients include a total PSA of more than 20 ng/ml, the clinical stage later than T1c, or the Gleason score of more than 7. More cases and prospective studies are required in the future. Varambally et al. revealed that UBASH3B mRNA expression was significantly differed between primary prostate cancer sites and metastatic sites (13), the same expression pattern has been observed in prostate cell lines. It was suggested that UBASH3B could drive prostate cancer metastasis, so the mechanism of tumor metastasis that is associated with UBASH3B may be our next investigation. Previous studies have shown that UBASH3B is involved in the regulation of T cell receptor signaling in activated T lymphocytes and regulates IFN-α-induced B cell autophagy (8). These altogether suggested UBASH3B might participate in immune response. The lncRNA-miRNA-mRNA network is involved in a number of prognostic-related genes, which also play a vital role in immune response, such as PIK3CG (14, 15) and BIRC3 (16, 17). In our study, we performed GSEA to explore the hallmarks of gene sets in high UBASH3B expression group and identified 18 up-regulated gene sets and 1 down-regulated gene sets such as “KRAS signaling up,” “inflammatory response,” “IL6 JAK STAT3 signaling,” and “oxidative phosphorylation.” The results of PPI network and TIMER indicate that UBASH3B may directly bind to LCP2, which is a gene involved in the inflammatory response. In addition, PIK3CG and BIRC3 were also found in PPI network and correlated with immune cells infiltrating in tumor microenvironment.

In recent years, the microenvironment involving many cell clusters (such as immune cells, tumor cells, and fibroblasts) in cancer is a hot issue. How UBASH3B participate in tumor microenvironment and influence tumor-infiltrating immune cells in prostate cancer should be explored. Based on the expression level of UBASH3B, we found six types of tumor-infiltrating immune cells in prostate cancer tissues, including B cells, CD4^+^ T cells, CD8^+^ T cells, macrophages, neutrophil, and dendritic cells. The infiltration of immune cells were found to be positively correlated with UBASH3B. Moreover, LCP2, PIK3CG, and BIRC3 were also positively correlated with these six types of immune cells in prostate cancer. CIBERSORT analysis assessed the estimated fraction of tumor-infiltrating immune cells in prostate cancer based on differentially expressed UBASH3B. There were 11 types of immune cells, including in naïve B cell, memory B cells, resting CD4^+^ memory T cell, activated CD4^+^ memory T cell, regulatory T cell, activated NK cell, M2 macrophages, resting dendritic cells, activated dendritic cells, resting mast cells and neutrophils, infiltrate in prostate cancer. These data suggest that UBASH3B expression levels are closely related to PCa-infiltrated immune cells, and further studies will be interesting to explore specific mechanisms of UBASH3B regulating these tumor-infiltrating immune cells in PCa. The crosstalk between UBASH3B and tumor-infiltrating immune cells in PCa suggests a potential target of UBASH3B for future treatment of PCa.

Since this study is based on preliminary data and hypothesis-generating predictions, there are some limitations in this study. First, the sample size in the cohort study of prostate cancer for IHC is limited and more precise data could be obtained with a larger number of samples. Second, the regulatory subnetwork of UBASH3B needs to be validated. In addition, the mechanisms of UBASH3B to regulate the infiltration of immune cells is required for further study.

In conclusion, our study has indicated that UBASH3B may represent a potential biomarker that contributes to poor prognosis for prostate cancer. UBASH3B may also play a vital role in the microenvironment of prostate cancer through regulating tumor-infiltrating of immune cells, suggesting UBASH3B as a therapeutic target to modulate anti-tumor immune response.

## Data Availability Statement

Publicly available datasets were analyzed in this study. This data can be found here: https://portal.gdc.cancer.gov/projects/TCGA-PRAD.

## Ethics Statement

This study was carried out in accordance with the recommendations of guidelines, the ethical committee of Second Xiangya Hospital, Central South University, China, with written informed consent from all subjects. All subjects gave written informed consent in accordance with the Declaration of Helsinki. The protocol was approved by the ethical committee of Second Xiangya Hospital, Central South University, China.

## Author Contributions

ZW and MP designed the study, acquired the data, and wrote the manuscript. ZW reviewed the manuscript. YW and LY contributed to the harvesting of the specimen. MP and LY supervised the study.

### Conflict of Interest

The authors declare that the research was conducted in the absence of any commercial or financial relationships that could be construed as a potential conflict of interest.
